# Editorial: Gut microbiota in health and disease

**DOI:** 10.3389/fgstr.2025.1568509

**Published:** 2025-02-25

**Authors:** Diogo Alpuim Costa, Pedro Barata Coelho, Conceição Calhau, Ana Faria

**Affiliations:** ^1^ Oncology Functional Unit, Hospital de Cascais, Cascais, Portugal; ^2^ Haematology and Oncology Department, CUF Oncologia, Lisbon, Portugal; ^3^ NOVA Medical School (NMS), Faculdade de Ciências Médicas (FCM), Universidade NOVA de Lisboa (UNL), Lisbon, Portugal; ^4^ Faculdade de Ciências da Saúde (FCS-UFP), Universidade Fernando Pessoa (UFP), Oporto, Portugal; ^5^ Instituto de Investigaçaão e Inovação em Saúde (i3S), Universidade do Porto (U.Porto), Oporto, Portugal; ^6^ Pathology Department, Centro Hospitalar Universitário do Porto (CHUP), Oporto, Portugal; ^7^ Centro de Investigação em Tecnologias e Serviços de Saúde (CINTESIS), Rede de Investigação em Saúde (RISE), NOVA Medical School (NMS), Faculdade de Ciências Médicas (FCM), Universidade NOVA de Lisboa (UNL), Lisbon, Portugal; ^8^ Unidade Universitária Lifestyle Medicine José de Mello Saúde, NOVA Medical School (NMS), Lisbon, Portugal; ^9^ Comprehensive Health Research Centre (CHRC), NOVA Medical School (NMS), Faculdade de Ciências Médicas (FCM), Universidade NOVA de Lisboa (UNL), Lisbon, Portugal

**Keywords:** microbiome, microbiota, dysbiosis, cancer, cardiovascular diseases, cerebrovascular diseases, treatment, toxicity

In recent years, terms such as microbiota and microbiome have gained prominence in pre- and clinical research, reflecting the growing understanding of its importance for human health and disease. But what is its meaning, and why does it attract so much attention?

The microbiota refers to the set of microorganisms, such as bacteria, fungi, viruses, and protozoa, living in a given environment, including the human body and, most importantly, the gastrointestinal tract (GIT). On the other hand, the microbiome represents not only the microorganisms *per se* but also their genetic material and interactions with the host organism (1, Almeida et al.).

The gut contains the largest population of microorganisms in the human body, with more than 100 trillion microorganisms and between 2 and 20 million microbial genes. These numbers correspond to approximately 200 g of body weight, the equivalent of a medium-sized mango. Thus, can we disregard more than half of our non-human cells (microbiota) and 99% of genes (microbiome) that coexist in our body? The microbiota composition is dynamic throughout life. It begins in intrauterine life with the transfer of bacteria from mother to fetus through the placenta, which appears to be definitively established by 3-4 years of age. With aging, microbiota enters a less diversified and stable state (1, Almeida et al.).

The healthy GI microbiota comprises more than 160 species of bacteria, of which Firmicutes and Bacteroidetes phyla represent more than 90%. Firmicutes are mainly composed of *Lactobacillus*, *Bacillus*, *Clostridium*, *Enterococcus*, and *Ruminococcus* genera; Bacteroidetes are composed of the *Bacteroides* and *Prevotella* genera. Other phyla include Actinobacteria, Proteobacteria, Fusobacteria, and Verrucomicrobia (Serpa).

As aforementioned, a set of bacteria is common to all healthy humans. However, like a fingerprint, the microbiota is unique to each individual, being influenced by several modifiable factors (e.g., breastfeeding, eating habits, lifestyle, and antibiotics) and non-modifiable factors (e.g., genetics, GIT anatomy, gestational age, type of delivery, and aging). Regarding modifiable factors, gut microbes can influence human health and disease by metabolizing substrates from the diet and host to produce bioactive compounds, including signaling compounds, biological precursors, and toxins (Zhang et al.). For instance, as pointed out by Silva et al., (Almeida et al., and Zhang et al., everyday dietary components are metabolized by the gut microbiota to produce metabolites (e.g., the transformation of choline, lecithin, and carnitine, found in red meat, eggs, fish, and dairy products, into trimethylamine – TMA – and then into trimethylamine N-oxide – TMAO- through gut microbiota metabolism and liver oxidation, respectively) that have been associated with atherosclerosis, arterial hypertension, heart failure, and cerebral infarction (CI).

Furthermore, Silva et al. discussed several gut microbiota-diet interactions. A diet rich in saturated fatty acids and sweet and salty foods modifies the gut microbiota, causing elevated levels of lipopolysaccharides (LPSs) in the circulation, leading to a pro-inflammatory state (metabolic endotoxemia). Conversely, some foods have a positive effect on the gut microbiota, for example, those that elevate short-chain fatty acids (SCFAs) production and the abundance of *Lactobacillus* and *Bifidobacterium* and those that are included in the Mediterranean diet, such as olive oil. Fermented foods, wine and beer, and coffee consumption also positively affect the gut microbiota composition.

From another perspective and still considering the modifiable factors of the gut microbiota, Nobre and Costa evaluated the importance of socioeconomic factors that may affect gut microbiota composition and, thus, influence health and disease status – a new term called “sociobiome”. Moreover, in a Dutch study, children residing in urban environments showed a lower abundance of *Bacteroides* and *Alistipes* than those in rural backgrounds. Conversely, in a Mexican study, the microbiota (*Prevotella copri* – *P. copri*, *Faecalibacterium prausnitzii*, *Rothia muciliginosa*, *Bifidobacterium* spp., and *Mitsuokella*) of children in rural areas had more anti-inflammatory characteristics that may enhance the microbiota resilience and decrease disease susceptibility.

Yet, another factor that remains largely overlooked is the significant diversity of the microbiota in the several subsections of the GIT. Serpa reinforced that different microenvironmental conditions control microbiota representativeness and density, namely, acidity, oxygen availability, the presence of antimicrobial compounds, and the time of transit through the GIT. In addition, the microbiota load increases from the stomach to the colon, creating a complex microbial ecosystem. Several studies describe sample collection from only the “small intestine” or “large intestine.” Lawal et al. highlighted evidence of the different microbiota communities of intestinal sub-organs in healthy individuals. These authors emphasized that the microenvironment of the small intestine is less favorable for microbial growth than the colon due to the lower pH, increased concentration of oxygen, and antimicrobial peptides produced by host cells of the epithelial lining of the small intestine. As such, most microbes in the small intestine are fast-growing, facultative anaerobes. Regional differences are particularly noticeable when comparing the segments of the colon because microbial diversity progressively increases from the proximal to the distal colon. The colon is a more conducive habitat for microbiota growth than the small intestine because it has a longer transit time and higher pH, a lower cell turnover, a lower redox potential, and fewer antimicrobials. In this microenvironment, many bacteria in the colon are fermentative, polysaccharide-degrading anaerobes.

Thus, the gut microbiota plays a crucial role in human health and disease. These microorganisms influence not only the digestion and absorption of macro- and micronutrients but also the synthesis of metabolites essential to homeostasis, the modulation of the immune system, and even the ability to influence behavior and mood.

At a global level in the adult population, the group of significant diseases responsible for the most morbidity and mortality each year includes cardio and cerebrovascular diseases, all types of cancer, respiratory diseases (mainly infections), and mental and substance use disorders. Nevertheless, the epidemiology varies significantly across the world. For instance, in low-income countries, communicable diseases tend to rank much higher. This starkly contrasts with high-income countries, where communicable diseases may not be in the top ten, and instead, cardiovascular disease and cancers tend to contribute the largest burden (2).

Mitigating and overcoming this dismal reality is crucial worldwide. In the last few years, we have been trying to investigate better the role of environmental and host microbiota in health and disease. Understanding the cause or consequence of this situation and how to maintain or restore the composition of the gut microbiota will be very helpful in developing new preventive and therapeutic avenues.

Recent studies prove that the balance between the microbial species in the gut microbiota is fundamental for maintaining the body’s homeostasis. Dysbiosis, an imbalance (altered abundance and diversity of microbiota) in the so-called healthy microbial community, can lead to increased intestinal permeability, the emergence of opportunistic microorganisms, chronic inflammation, metabolic alterations, and an unfavorable shift in the response of the innate and acquired immune systems. A growing body of proof suggests that dysbiosis is a hallmark of intestinal and several extra-intestinal diseases, such as cardiovascular and neurological disorders, cancer, and many others (1, Silva et al.; Almeida et al.; Serpa; Zhang et al.; Nobre and Costa; Lawal et al.).

In this context, we highlight some of the main points that were explored in this Research Topic that focused on the association between microbiota and different health and disease processes:

## 1 Immune system modulation

The commensal microbiota has been implicated in regulating a wide range of physiological processes within the GIT and at distant tissue sites. This “external metabolic organ” interacts with the human innate and adaptative immune systems.

Microbial factors, such as virulence factors and microbe-associated molecular patterns (MAMPs), are primarily responsible for modulating the immune response. Duarte Mendes et al. and Yu et al. reviewed the immune-microbiota cell-cycle crosstalk, using colorectal cancer (CRC) pathogenesis as an explanation model for immune evasion, cancer cell survival, tumor microenvironment modulation, and metastases. The lamina propria beneath the epithelial cells (IECs) harbors immune cells, encompassing the gut-associated lymphoid tissue (GALT), including antigen-presenting cells such as dendritic cells, T cells, and B cells. The several pattern-recognition receptors (PRRs), such as toll-like receptors (TLRs), expressed in IECs and immune cells are thought to recognize MAMPs of commensal bacteria. Thereafter, the dendritic cells are activated by the microbes or by microbe-derived elements (e.g., metabolites, products) via interactions with PRRs. When activated, they travel to the mesenteric lymph nodes and orchestrate the differentiation of naïve T cells into effector T cells, mainly Tregs and helper 17 (Th17). A subset of these cells may migrate back to the intestine or enter the systemic circulation, thus locally and systemically modulating the host’s immune system. Additionally, MAMPs or microbe metabolites can also stimulate the immune system through other mechanisms, including stimulation of enteric neurons with the release of neurotransmitters that regulate the immune cell function, secretion of immunoglobulin (namely IgA), and activation of the innate immune response.

Gut microbiota can exert beneficial or detrimental effects on immune response by producing metabolic products and signaling molecules, which influence diverse functions in different organs.

Among these bacteria-derived metabolites, SCFAs have been shown to have several beneficial effects on the organism. As addressed by Serpa, the SCFAs derived from carbohydrates and amino acid fermentation are the most relevant end product to be absorbed in the human gut and used by other bacteria. The most abundant SCFAs are acetate, propionate, and butyrate. SCFAs regulate gut pH, impact the metabolic functions of invasive pathogens, inhibit their growth and reproduction, and suppress the expression of virulence genes in pathogens. The regulatory role of SCFAs in the innate immune system includes pyrin dome-containing protein 3 (NLRP3) inflammasome, receptors of TLR family members, neutrophils, macrophages, natural killer cells, eosinophils, basophils, and innate lymphocyte subsets. The regulatory role of SCFAs in the adaptive immune system includes T-cell subsets, B cells, and plasma cells. Yu et al. described one of the putative anti-inflammatory mechanisms of SCFAs, mainly through butyrate, that involves an enhancement of CD8+ T cell metabolism and their differentiation into memory T cells.

Many members of the gut microbiota are able to produce SCFAs in the colon. *Akkermansia muciniphila* (*A. muciniphila*) is recognized as a key element for producing these metabolites. In a seminal paper, Iwaza et al. reviewed the state of the art of this particular species belonging to the Verrucomicrobia phylum. In 2004, Derrien et al. (3) discovered and isolated *A. muciniphila* from the stool of a healthy individual. *A. muciniphila* relies on mucin for carbon, nitrogen, and energy. The capacity of this bacteria to degrade and use mucin as a unique source of carbon and nitrogen gives it significant importance in the human GIT, allowing other bacteria to survive and grow by using the metabolites resulting from mucin degradation. SCFAs also play a role in the inflammatory status of the host, regulating the immune system and improving the gut barrier function (3).

SCFAs have been demonstrated to be relevant in several pathologies. In CRC, an increased abundance of pathogenic microbes, such as *Fusobacterium nucleatum* (*F. nucleatum*), and a decreased abundance of butyrate-producing bacteria have been observed, resulting in lowered SCFAs levels and enhanced inflammation (Yu et al.). In hypertension, lowered butyrate-producing gut microbial counts and deficient intestinal absorption of SCFAs have been observed (Almeida et al.). Koester et al. also pointed out that *F. nucleatum*, linked to CRC progression and metastasis, has been associated with CpG island methylator phenotype in the female sex. Furthermore, this group investigated the ambivalent role of RET as an oncogene or tumor suppressor in CRC. Their study offered a proof-of-principle that CRC risk-modulating gut microbial effects depend on sex and genetics, and they underscored the importance of evaluating sex as a biological variable in research and of reporting the sexes of both human and non-human study participants.

Zhu et al. reported another example of the anti-inflammatory potential of SCFAs. Their pioneering study found that serum levels of stress-inducible 72-kDa heat-shock protein (HSP72) and zonulin, immunomodulatory and anti-inflammatory proteins, were increased in patients with CI. Accordingly, the upregulation of these proteins was related to specific gut microbiota alterations and the clinical severity of CI. Moreover, the abundance of bacteria *Eubacterium fissicatena* (*E. fissicatena*) and *E. eligens* groups and *Romboutsia* manifested a remarkably positive correlation with serum HSP72. The abundance of bacteria *E. fissicatena* group and *Acetivibrio* had a significantly positive correlation with zonulin levels. The genus *Eubacterium* has been identified to contribute to massive aspects of human health, for most of the family produce SCFAs, especially butyrate.

Nevertheless, the other side of the coin can also happen, and some microbiota-derived metabolites have been linked to an increased risk of certain diseases. TMAO appears to be correlated with cardio and cerebrovascular diseases. (Almeida et al., Zhang et al.) Indeed, bacterial translocation from the gut to the heart and the discovery of bacterial DNA in atherosclerotic plaques led to the gut being considered a potential reservoir of opportunistic microorganisms. According to the narrative review of Almeida et al., relevant data from 19 prospective studies reported that higher levels of TMAO and its precursors were associated with a higher risk of major adverse cardiovascular events and all-cause mortality. Also, there appears to be a graded association between TMAO levels and the risk of subsequent cardiovascular events in patients with recent ischemic stroke. These TMAO inflammatory signals involve NF-κB, NLRP3 inflammasome, MAPK/JNK pathway, and gut microbiota modulation. Although, according to Zhang et al.’s systematic review, which included six studies of acute ischemic stroke and one study of intracerebral hemorrhage, there is limited evidence indicating that high baseline plasma levels of TMAO may be associated with poor IC outcomes.

Moreover, based on the potentially predictive risk of gut microbiota for cardiovascular disease, Almeida et al. and Silva et al. reviewed data describing that there could be an association between leaky gut and higher levels of LPSs in the bloodstream. These endotoxins are released when gram-negative bacteria die and lyse, releasing their content into the surrounding environment. Therefore, LPSs and their derivates act as MAMPs and induce acute and chronic inflammatory responses when entering the bloodstream, as the immune system recognizes these active substances as foreign invaders. Furthermore, Almeida et al. emphasized that gut microbiota can also affect the host’s insulin resistance, glucose metabolism, and certain hormone levels, such as leptin and ghrelin, which can lead to increased inflammation or regulate appetite, leading to atherosclerosis.

Still, within the scope of metabolites with potentially harmful effects on health, Serpa described the role of cysteine in microbiota and human cells crosstalk, favoring cancer. Cysteine is a very relevant compound in cancer metabolism that constitutes the main thiol in the biological fluids of cancer patients, which comes from endogenous synthesis, transsulfuration pathway, and protein degradation or by increased intestinal absorption of cysteine intestinal content that originated from diet and microbiota metabolism. In some types of cancer, this amino acid was shown to be a relevant carbon source, sustaining bioenergetics and biosynthesis, and a pivotal source needed for ATP production, cell cancer survival, and disease progression. Moreover, cancer cells that exhibit metabolic dependence on cysteine account for increased glutathione levels and scavenging capacity of reactive oxygen species to cope with oxidative stress, contributing to better antioxidant potential.

These findings reinforce data indicating that microbiota could modulate directly or indirectly immune processes in specific individuals, potentially influencing the predisposition to the risk of some diseases and their clinical course.

## 2 Microbiota disease signatures

There is an accumulation of evidence that the human gut microbiota plays a role in maintaining health and that dysbiosis is associated with risk for many communicable and non-communicable diseases. Furthermore, microbial signature taxa are being identified for the diagnosis of some diseases, like ulcerative colitis, Crohn’s disease, irritable bowel syndrome, depression and anxiety disorders, auto-immune disorders, cancer, and COVID-19 infection, among others.

Lawal et al. highlighted evidence about the variations in the composition of the microbiota communities identified at specific sites along the GIT in healthy individuals and patients with inflammatory bowel diseases (IBD), ulcerative colitis, and Crohn’s disease, which are characterized by persistent inflammation and gut damage. IBD patients have different microbiota than healthy individuals (e.g., in the duodenum, the beneficial genera of bacteria *Bifidobacterium* and *Lactobacillus* are notably decreased in IBD, whereas the populations of *Bacteroides* and *Escherichia* genera are increased).

In their review, Bibbó et al. explored the role of the gut-brain axis in depression and anxiety disorders. Indeed, gut microbes can interact with the brain, interfering with behavior through mechanisms such as amino acid metabolism, SCFAs, vagus nerve, endocrine signaling, and immune responses. For instance, a systematic review showed that about 50 bacterial taxa exhibit differences between patients with major depressive disorders and controls.

Finally, several studies have shown that cancer patients often experience changes in the composition of their gut microbiota compared to healthy individuals (1, 4). These changes may be associated with an increased risk of developing cancer.

As earlier mentioned, specific intestinal pathogens, such as *F. nucleatum* or colibactin-producing *Escherichia coli*, are associated with CRC (1–Yu et al.). Chen et al., through Mendelian randomization (MR) analysis, investigate causal associations between gut microbiota and intrahepatic cholangiocarcinoma (ICC), an aggressive liver cancer with a poor prognosis. Genetically predicted increases in *Veillonellaceae*, *Alistipes*, *Enterobacteriales*, and Firmicutes were suggestively associated with higher ICC risk, while increases in *Anaerostipes*, *Paraprevotella*, *Parasutterella*, and *Verrucomicrobia* appeared protective. Bioinformatics analysis revealed that differentially expressed genes near gut microbiota-associated loci may influence ICC through regulating pathways and tumor immune microenvironment.

Parallelly, the gut microbiota has been significantly associated with differentiated thyroid cancer (DTC). However, the causal relationship between the gut microbiota and DTC remains unexplored. Thus, Hu et al. investigated the causal relationship between the gut microbiota and DTC. In this context, four bacterial traits were associated with the risk of DTC (class Mollicutes, phylum Tenericutes, genus *Eggerthella*, and order Rhodospirillales). Additionally, four other bacterial traits were negatively associated with DTC (genus *E. fissicatena* group, genus *Lachnospiraceae* UCG008, genus *Christensenellaceae R-7* group, and genus *Escherichia Shigella*).

Observational epidemiological studies suggested an association between the gut microbiota and breast cancer (BC). Still, it remains unclear whether the gut microbiota causally influences the risk of BC (1, 4). Zhang et al. employed a two-sample MR analysis to investigate this association. The inverse variance-weighted (IVW) MR method examined the causal relationship between the gut microbiota and BC and its subtypes. The IVW estimates indicated that an increased abundance of genus *Sellimonas* was causally associated with an increased risk of estrogen receptor-positive (ER+) BC, whereas an increased abundance of genus *Adlercreutzia* was protective against ER+ BC. For human epidermal growth factor 2 positive (HER2+) BC, an increased abundance of genus *Ruminococcus2* was associated with a decreased risk, whereas an increased abundance of genus *Erysipelatoclostridium* was associated with an increased risk. In a case report, Vilhais et al. described the longitudinal analysis of the gut microbiota of an ER+/HER2- BC patient throughout the therapeutic approach with a 6-month regimen of endocrine therapy (ET) plus a CDK 4/6 inhibitor (CDK4/6i). This clinical case evidenced a shift in gut microbial dominance from Firmicutes to Bacteroidetes primarily due to a noteworthy increase in the relative abundance of *P. copri* following the treatment course. *P. copri* is an abundant member of the human gut microbiota, whose relative abundance has curiously been associated with positive and negative impacts on several diseases, alongside some pharmacomicrobiomic implications. The link between *P. copri* and different types of cancer remains inexplicable. However, some hypothesize that *Prevotella* genera may be involved in breast disease due to its estrogen-deconjugating enzymatic activity. The role of *P. copri* and other bacterial species capable of metabolizing estrogens in BC, called “estrobolome”, is particularly interesting for future research (1, 4).

Parallel to what was observed at the gut level, it also appears that there are specific local microbiota signatures for each type and subtype of cancer. These findings result from a close relationship between the intestine and the primary tumor and/or an environment conducive to the growth of microorganisms at the tumor microenvironment level. Is there a tumor-gut axis? (1, Yu et al., Vilhais et al. 5) In this sequence, Vilhais et al. showed in the analysis of the local microbiota of the breast surgical specimen an interestingly high dissimilarity between the residual tumor and respective margins, suggesting markedly different microbial compositions. While the margins revealed a more diverse distribution of microbial species, the tumor’s microbial composition was dominated by fewer species, particularly *Streptococcus pneumoniae* and *Atopobium vaginae*. Additionally, the authors described the data of a preclinical study reporting that *Streptococcus* in BC cells can inhibit the RhoA-ROCK signaling pathway to reshape the cytoskeleton and help tumor cells resist mechanical stress in blood vessels, thus promoting hematogenous metastasis.

## 3 Pharmacomicrobiomics

Finally, inter-individual heterogeneity in drug response is a serious problem that affects the patient’s well-being and poses enormous clinical and financial burdens on a societal level. Understanding the role of the gut microbiota in drug response may enable the development of microbiota-targeting approaches that enhance drug efficacy and decrease toxicity. Pharmacomicrobiomics is an emerging field investigating the interplay of microbiota variation and drug response and disposition (absorption, distribution, metabolism, and excretion). Modulating the gut microbiota has the potential to become a very attractive approach to managing drug efficiency toward more personalized medicine (1, 6).

Manipulating the gut microbiota through diet, biotics, or fecal transplantation (FMT) is being investigated as a potential strategy for several diseases. For instance, as Silva et al. mentioned, microbiota-dependent SCFAs production can be enhanced by consuming high-fiber diets such as the Mediterranean one. In a pre-clinical study, Nguyen et al. investigated the effect of an exopolysaccharide (EPS) probiotic molecule produced by the commensal bacterium *Bacillus subtilis* (*B. subtilis*) on BC phenotypes. Although *B. subtilis* is commonly included in probiotic preparations and its EPS protects against inflammatory diseases, it was virtually unknown whether *B. subtilis*-derived EPS affected cancer. Short-term treatment with EPS inhibited the proliferation of specific BC cells, while more extended treatment in mice led to tumor growth. Additional experiments are needed to determine the physiological relevance of EPS on BC, and a favorable risk-benefit ratio is warranted to be implemented in clinical practice.

Silva et al. also addressed a hot topic among researchers and clinicians, the FMT. This procedure is an established treatment for recurrent *Clostridioides difficile* infections (CDIs). Furthermore, FMT is indicated for patients with multiple recurrences of CDI for whom appropriate antibiotic treatments have failed, and it has cure rates of 80%–90%. In addition, it seems promising as a treatment for many other conditions, like IBD, obesity, metabolic syndrome, psychiatric neurological diseases, COVID-19, and cancer (1, 2, 7, Almeida et al., Duarte Mendes et al., Yu et al., Bibbó et al.). This procedure consists of collecting feces from a healthy donor and introducing them into a patient’s GIT to treat a certain disease linked with the alteration of the gut microbiota. FMT can be performed through the upper GIT, via a duodenal tube or capsules taken orally, or through the lower GIT via colonoscopy or an enema. The authors discussed the importance of including the dietary patterns of stool donors and receptors in the stool donor screening process and the importance of monitoring receptors’ diet to ensure the engraftment and success of the FMT (Silva et al.).

Regarding toxicity, drug–microbial interactions can be categorized into two classes: microbiota modulation of toxicity (MMT) and toxicant modulation of the microbiota (TMM). MMT refers to transforming a drug (chemical) by microbial enzymes or metabolites to modify the chemical in a way that makes it more or less toxic. TMM is a change in the microbiota that results from chemical exposure. An example of MMT could occur by the induction of host-detoxifying enzymes by microbial metabolites that shift the metabolic pathway for a chemical and result in differential toxicity levels (6). Gonçalves-Nobre et al. reviewed some mechanisms, including the irreversible dose-dependent anthracyclines cardiotoxicity related to oxidative stress and the reversible cardiotoxicity with trastuzumab in BC treatment. The authors highlighted that altered gut microbiota composition has been linked to long-term cardiotoxicity. *Bacteroides* spp., *Coriobacteriaceae UGC-002*, and *Dubosiella* have deleterious effects on the myocardium, mainly due to the promotion of inflammation. On the other hand, *Alloprevotella*, *Rickenellaceae RC9*, *Raoultella planticola*, *Klebsiella pneumoniae*, and *E. coli BW25113* can induce cardioprotection predominantly by increasing anti-inflammatory cytokines, promoting intestinal barrier integrity and early metabolization of doxorubicin.

The relationship between microbiota, health, and disease is complex and multifaceted. It involves interactions between microorganisms, inflammatory processes, metabolism, and immune responses. More research is needed to elucidate better these mechanisms, identify optimal interventions, and determine their efficacy and safety in different clinical settings.
